# Effect of 4 Weeks of Cervical Deep Muscle Flexion Exercise on Headache and Sleep Disorder in Patients with Tension Headache and Forward Head Posture

**DOI:** 10.3390/ijerph18073410

**Published:** 2021-03-25

**Authors:** Wonho Choi

**Affiliations:** Department of Physical Therapy, Gachon University, Incheon 21936, Korea; whchoi@gachon.ac.kr; Tel.: +82-32-820-4423; Fax: +82-32-820-4420

**Keywords:** deep muscle flexion exercise, stretching exercise, tension headache, headache disorder, sleep disorder

## Abstract

The purpose of this study was to investigate the effect of flexion exercise of the deep cervical muscles on headache and sleep disorders in patients with tension headaches and forward head posture. A total of 32 patients with tension headaches and forward head posture were randomly assigned to two groups: an experimental group (*n* = 16) and a control group (*n* = 16). The experimental group performed cervical deep muscle flexion exercises for 4 weeks, whereas the control group performed stretching exercises for the same period. The Henry Ford Hospital Headache Disability Inventory (HDI) was used for headache assessment, and the Korean version of the Pittsburgh Sleep Quality Index (PSQI-K) was used for sleep disorder assessment. The experimental group showed a significant reduction in both HDI and PSQI-K score after 4 weeks of intervention (*p* < 0.001), while no significant difference was found in the control group (*p* > 0.05). On comparing the experimental and control groups, we found a significant difference in changes in the HDI and PSQI-K between the groups (*p* < 0.05). The results indicate that flexion exercise of the deep cervical muscles in patients with tension headache and forward head posture will improve the quality of life and activities of daily life by mitigating headaches and sleep disorders.

## 1. Introduction

There is a high prevalence of cervical disease today as the lifestyles of modern people have changed; this subsequently poses a high economic burden [1]. The prevalence may be due to the excessive use of body parts and muscles because of the repetitive and monotonous work activities we perform today [2]. Unlike in the past, the use of various computers and smartphones has increased widely in various fields such as production, management, and communication [3], causing serious difficulties related to the neck due to an improper posture [4,5].

Forward neck posture is a phenomenon in which the head is moved forward in the sagittal plane of the cervical spine, and the head is placed in front of the trunk or turns [6,7]. Therefore, structurally, the central line of the head moves forward and upward, increasing the weight of the head supported by the neck. The craniocervical region compensates with a slight distortion. Over time, this posture results in shorter adaptability in the suboccipital muscles, posterior ligaments, and membranes associated with the atlanto-axial and atlanto-occipital joints [8]. This causes an increase in lordosis and shortening of the occipital muscles. It further causes abnormal and continuous muscle contraction and relative compensatory action of the muscles of the occipital bone, neck, and shoulders, resulting in a change in the shape of the cervical lordosis [9]. Eventually, the forward neck posture causes muscle weakness in the deep flexors of the cervical spine, which not only reduces neck stability but also causes pain [10,11,12]. Deep muscles, such as the longus colli and longus capitis, are less active than the superficial muscles, such as the sternocleidomastoid and anterior scalenus [13].

A headache is defined as pain occurring in the head and neck and is a common disease experienced by 96.7% of the global population during their lifetime [14]. The inconvenience and social costs caused by headaches go beyond the problems of individual patients and their families and increase the burden on the society as a whole [15]. Head forward posture is known to cause tension headaches and even sleep disorders [10,11,12]. Tension headaches are caused by ischemia in the upper cervical region because the muscle tone of the back of the cervical bones is consistently high [16]; the cervical nerve roots of C1 to C3 are weakened, resulting in the release of harmful irritating tissues to the joint capsule, ligaments, and spine [16]. Headaches caused by abnormal muscle tone around the skull and cervical spine are known as tension headaches [17]. In addition, in people with tension headaches, an increase in forward head posture and a decrease in neck movement are specific and clinical [18]. Tension headaches cause sleep disorders, which are related to quality of life and lead to disabilities [10]. Although the mechanism has not been clearly identified, sleep disorders and headaches are closely related [19]. Among the types of headaches that cause sleep disorders, tension headache is a representative disorder [20]. Therefore, in order to solve the problems associated with forward head posture, various approaches such as stretching, muscle strengthening exercises, and manual therapy are being attempted [21].

In a previous study by Lynch [21], it was reported that muscle strengthening and stretching exercises were effective for the forward head posture. Studies comparing the effectiveness of manual therapy with flexion exercise to improve the forward head posture have also been conducted [22]. In addition, studies have reported the effectiveness of strengthening exercises of the deep flexor muscles of the cervical area for correct posture and relief of tension headache and for improving forward head posture [7]. The method used in these studies is an exercise program that strengthens the deep muscles that play a major role in the stability of the spine; thus, the position of the head moved forward can be moved backward [23,24]. This is a method for activating the deep flexors, longus colli, and longus capitus by minimizing the activation of the superficial muscles, such as the sternocleidomastoid and anterior scalenus muscles [24]. In a current study on the functional specificity of the superficial and deep muscles of the neck, it has been noted that interventional methods for patients with forward head posture have become systematic; the therapeutic effect and importance of these methods have been emphasized as it relieves physical problems in humans [25,26].

Therefore, the purpose of this study was to investigate the effect of deep neck flexion exercise on headache and sleep disability in patients with tension headaches due to forward head posture.

## 2. Materials and Methods

### 2.1. Ethical Approval

This study was approved by the Institutional Review Board of Gachon University (1044396-201710-HR-167-01 and 17 October, 2017F). All participants signed a statement regarding informed consent before beginning the study.

### 2.2. Participants

Thirty-two subjects with tension headaches caused by forward head protraction participated in this study. The inclusion criteria were as follows: craniovertebral angle <49° [27] and constant or intermittent neck pain within the last 3 months. The exclusion criteria were as follows: neurological or vascular problems, orthopedic problems in the neck or shoulder region, and a history of spine surgery.

### 2.3. Procedures

The study design was a pretest–posttest control group design. The 32 subjects who met the selection criteria were randomly assigned to two groups, 16 subjects in the experimental group (EG) and 16 subjects in the control group (CG), using a random number table generated by the Microsoft Excel 2010 program. As an intervention method, the EG performed cervical deep muscle flexion exercises and the CG performed stretching exercises. The Henry Ford Hospital Headache Disability Inventory (HDI) and Korean version of the Pittsburgh Sleep Quality Index (PSQI-K) were measured at baseline and 4 weeks after the intervention. One practice session was conducted before the evaluation so that the subjects could understand the experimental method. All values were measured three times, and the average value was recorded.

### 2.4. Intervention

#### 2.4.1. Cervical Deep Muscle Flexion Exercise

##### General Isometric Movement

The general isometric exercise consisted of two movements in stages by referring to the “Kinetic control book: The management of uncontrolled movement” written by Mark Comerford and Sarah Mottram [28].

**Figure 1:**
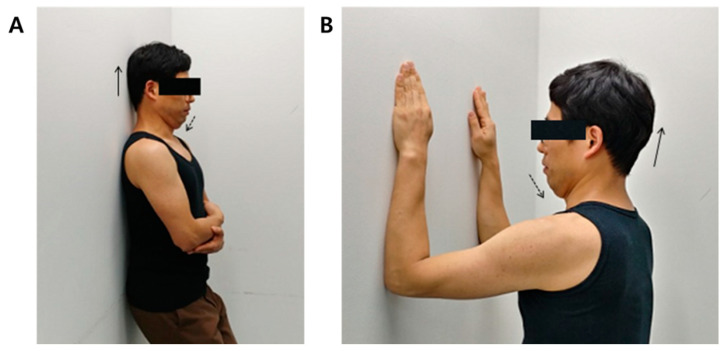
(**A**) General isometric movement Exercise 1. (**B**) General isometric movement Exercise 2.

Exercise 1 was carried out at weeks 1 and 2 of the 4-week exercise program for the subject to support the head and thoracic vertebrae against the wall and to maintain the neutrality of the shoulder and jaw joints. Independent isometric exercises of upper cervical flexion were performed before the lower cervical vertebrae started flexion to provide support and feedback (Figure 1A).

Exercise 2 was carried out at weeks 3 and 4 of the 4-week exercise program. The forearm was placed vertically on the wall and the scapula was kept in the middle position in a facing position, and the head was placed above and behind the shoulders. The independent upper cervical spine was maintained and slowly flexed only to perform isometric exercises. Similar to Exercise 1, the upper cervical spine maintained a neutral position of the scapula and the jaw joint until the lower cervical spine was not flexed and then flexed independently to perform isometric exercises (Figure 1B).

One set of 10 repetitions of 10 s per exercise was set, and the rest time was set to 5 s, and a total of three sets were performed. A 2-min break was allowed between sets [29].

##### Pressure Biofeedback Exercise

**Figure 2:**
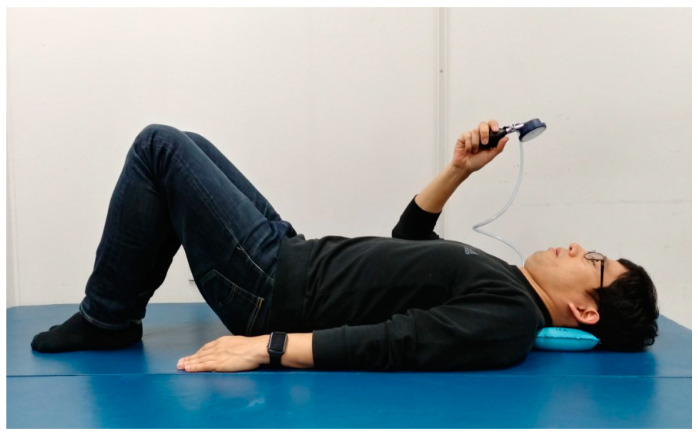
Cervical deep muscle flexion exercise.

The subject laid in a hook-lying position, with an air bag under the occipital region, and the chin pulled to compress the equipment; the pressure level on the instrument panel was visually checked. The pressure level is an average that allows the subject to contract the deep flexor muscles of the neck without feeling discomfort via a preliminary simulation (Figure 2). During the 4-week exercise program, the pressure was 22 mmHg at week 1, 24 mmHg at week 2, 26 mmHg at week 3, and 28 mmHg at week 4. The exercise method was maintained for 10 s at the target level, and the rest time was set as one set of 10 repetitions with 5 s. A total of three sets were performed, and the rest time between sets was 2 min [30].

#### 2.4.2. Stretching Exercise

The stretching exercise method was a self-stretching exercise reported by Evjenth and Hamberg [31] and was performed on the pectoralis major and minor muscles, levator scapular muscles, and upper trapezius, which were shortened owing to the muscle imbalance of the upper crossed syndrome.

##### Pectoralis Major and Minor Stretching

**Figure 3:**
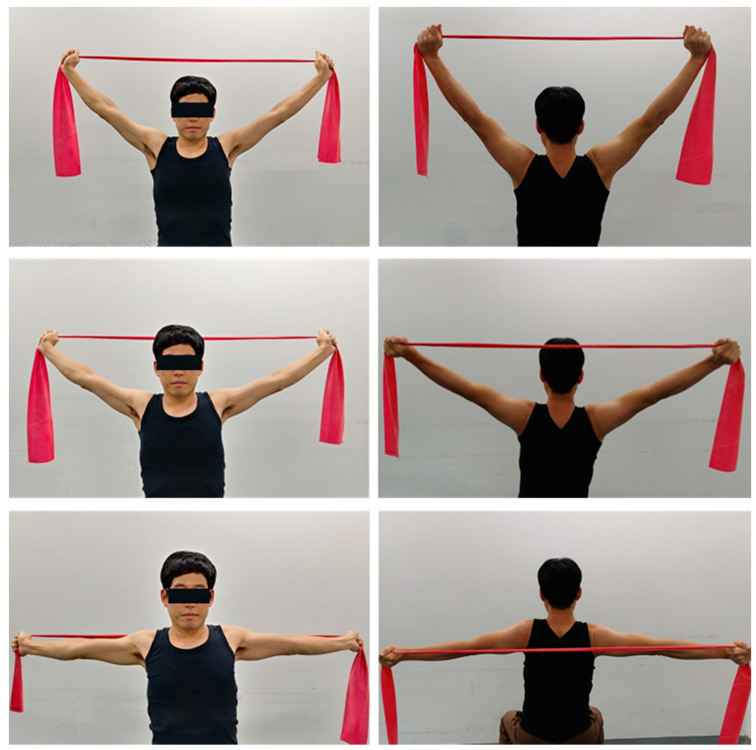
Stretching exercise of pectoralis major and minor.

The elastic band was held with hands so that the subjects could sit in a chair that could hold the seat underneath the seat and sit in a proper position by pulling the chin and extending the elbows, raising both arms above the head, and lowering to the back. The subjects were asked to close both hands, stretch their arms out with their hands still, pull them outward for 45 s, and then repeat this movement three times per cycle (Figure 3).

##### Levator Scapulae Stretching

**Figure 4:**
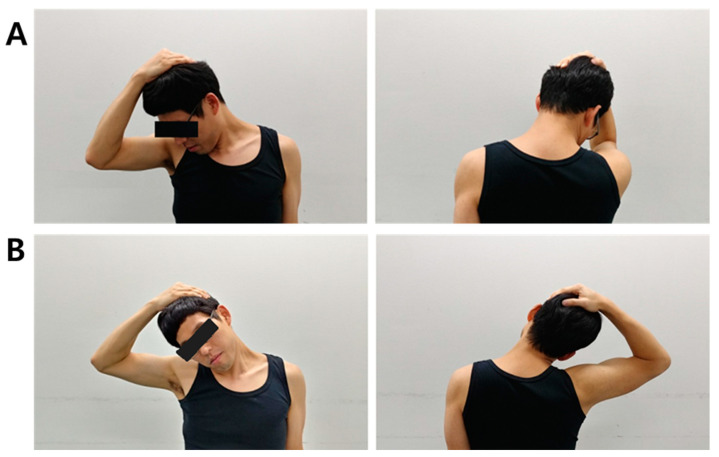
(**A**) Stretching exercise of levator scapular (**B**) upper trapezius.

The subjects sat in a proper position on a chair that held the seat underneath, raised the shoulder complex, and performed rotation on the same side with side bending of the ipsilateral side accompanied by cervical flexion. At the same time as the depression of the shoulder, they were asked to hold the side of the seat under the chair and fix it, perform side bending of the trunk, hold the right and left sides for 45 s, and repeat the movement three times per cycle (Figure 4A).

##### Upper Trapezius Stretching

The subjects sat in a proper position in a chair that held the seat underneath, raised the shoulder complex, and performed rotation on the opposite side with side bending of the ipsilateral side accompanied by cervical spine flexion. At the same time as the depression of the shoulder, they were asked to hold the side of the seat under the chair and fix it, perform side bending of the trunk, hold the right and left sides for 45 s, and repeat this movement three times per cycle (Figure 4B).

### 2.5. Measurements

#### 2.5.1. Craniovertebral Angle (CVA)

**Figure 5:**
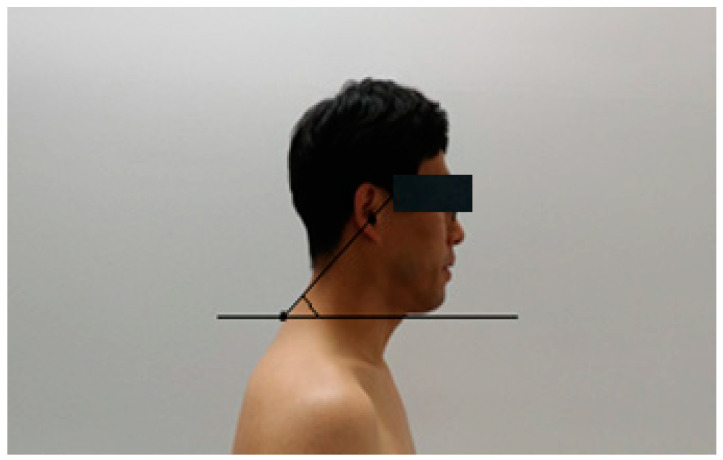
Measurement of craniovertebral angle.

The cranial vertebral angle was measured to screen the subjects. The cranial column allowed the subjects to sit on a fixed chair and look straight ahead, with their arms naturally on their knees and their neck in a position they were most comfortable in. Before the posture measurement, the subjects performed three repetitions of flexion and extension of the neck. Then, the horizontal line passing through the spinous process of the cervical spine, the line connecting the spinous process of the 7th cervical spine and the ear canal, and the angle formed by the intersection of the two lines were measured with a goniometer. According to a previous study, the head forward posture is less than 49°, and the smaller the cranial vertebral angle, the more advanced the head forward posture is (Figure 5) [27].

#### 2.5.2. Headache Disability Inventory

For the evaluation of headache, the HDI was used. This tool is a useful measure for evaluating the effects of headaches and treatment and the effect of headaches on daily life [32]. It consists of 25 questions, and one can choose from three scales. Four points for “yes”, 2 points for “sometimes”, and 0 points for “no”. The higher the total, the more severe the headache.

#### 2.5.3. Sleep Quality Index

Sleep disturbance was evaluated using the Korean version of the Pittsburgh Sleep Quality Index (PSQI-K) to evaluate sleep quality and insomnia [33]. This measure evaluates sleep quality and disability in the past month. This index comprised the following items: subjective sleep quality, sleep latency, sleep duration, habitual sleep efficiency, sleep disturbances, use of sleeping medication, and daytime dysfunction, which were measured with 0–3 points for each item, with a total score of 0–21 points. A score exceeding 5 indicated having a sleep disorder [34].

### 2.6. Sample Size Estimation

G power 3.0.1 software (Heinrich Heine University Düsseldorf, Düsseldorf, Germany) was used to determine the sample size. Based on our pilot study results, a total of 32 participants were estimated to be required with an effect size of f = 0.186, a significance level of 0.05, a power of 0.80, and a correlation among rep measures of r = 0.768 when a clinically significant interaction was observed between time points (two events: pre and post) and two groups.

### 2.7. Statistical Analysis

Data processing in this study was performed using SPSS for Windows version 18.0 (IBM, Armonk, NY, USA), and the measured values of all items were calculated as the mean and standard deviation (SD). All subjects were confirmed to be normally distributed through the Shapiro–Wilk normality verification. Chi-squared test and independent *t*-test were performed to compare the general characteristics of subjects with the homogeneity test between groups. Paired *t*-test was performed to compare the before and after differences according to the exercise method within the group, and an independent sample *t*-test was performed to compare the differences in dependent variables according to the exercise method between the groups. The level of statistical significance was set to α = 0.05.

## 3. Results

A total of 32 patients (19 men and 13 women) participated in this study. The general characteristics of the participants are presented in Table 1. No significant differences were found between the groups in the homogeneity test (*p* > 0.05).

### 3.1. Variations in Headache Disorder

There was significant reduction in HDI score after the intervention in the EG (64.56 ± 13.38 to 48.12 ± 10.78, *p* < 0.001), while no significant difference was found in the CG (57.88 ± 10.24 to 57.38 ± 9.98, *p* = 0.679). Additionally, there was a significant difference in the changes in the HDI score between the groups (16.44 ± 6.25 to 0.50 ± 4.75, *p* = 0.017) (Table 2).

### 3.2. Variations in Sleep Disorder

Similar to headache disorder, significant difference in PSQI-K was found in the EG (10.81 ± 1.97 to 7.56 ± 2.39, *p* < 0.001), while no significant difference was found in the CG (10.50 ± 2.03 to 10.00 ± 2.25, *p* = 0.072). There was a significant difference in the changes in PSQI-K between the groups (3.25 ± 1.29 to 0.50 ± 1.03, *p* = 0.002) (Table 2).

## 4. Discussion

Forward head posture causes considerable strain and musculoskeletal pain around the neck and causes tension headaches [35,36]. Tension headaches cause various problems in both work and daily life. In particular, they cause neck movement limitations and neck and shoulder pain [10]. In addition, they affect the comfort of daily life, such as sleep [37]. This study investigated the effect of performing deep cervical muscle flexion exercise for 4 weeks on tension headache and sleep disability in patients with forward head posture, and the following results were verified.

There was a significant decrease in the headache disorder index in the group with tension headaches and deep cervical muscle flexion exercises. In the forward head posture, the position of the head is moved forward, causing continuous contraction of the suboccipital muscles. The relatively high density of the muscle spines in the subocipital muscles plays an important role in providing neural biofeedback to the head position and for movement speed [38,39,40]. This increases the input of noxious receptor information and decreases the pain threshold through continuous stimulation of the caudate nucleus of the trigeminal nerve [41,42].

To treat tension headaches, the pain threshold is suppressed; it can be seen as a fundamental solution that the myofascial pain point is treated by elongating the shortened muscle according to the improvement of the forward head position. In addition, it has been reported that weakening of the muscle endurance of the cervical deep muscles is an important factor that causes tension headaches [43]. The exercise program used in this study helps activates the deep muscles of the neck and maintains the correct posture [13]. Correct posture recognition is possible by activating the deep muscles through the deep muscle flexion exercise.

Therefore, we believed that the cause of the reduction effect of tension headaches was the change in the craniospinal angle through the deep neck flexion exercise. This contributed to the reduction of the suboccipital muscle tension as the cervical spine rearranged and resulted in a reduction of the pain threshold.

The cervical deep muscle flexion exercise group showed a significant decrease in the sleep disorder index. It is important to assess sleep disturbances in patients with tension headaches. Previous studies on the relationship between sleep disorders and headaches have shown that sleep disorders can cause tension headaches, sleep headaches, and other migraine headaches; the most common sleep disorders are sleep apnea and insomnia. Since about half of the patients complained of headaches [44,45], it is estimated that if the headaches mitigate, sleep disorders will also improve. Since there are various causes such as alcohol, smoking, mental stress, traumatic stress, depression, and fatigue, in addition to headaches, that cause sleep disorders, further research on sleep disorders is necessary as it was not possible to determine the extent of its relationship with headaches in our study.

Finally, as a result of the comparison between groups in this study, the cervical deep muscle flexion exercise group showed a higher exercise effect on tension headaches and sleep disorder than the stretching EG. This is because the density of the muscle spindles is higher in the deep flexors of the neck than in other muscles [46]; therefore, we considere correct posture recognition to be possible by activating the deep flexors of the cervical area.

Therefore, it is presumed that the results obtained with the cervical deep muscle flexion exercise were more positive than those obtained with the stretching exercise due to the increase in spinal stability, decrease in superficial muscle tension, and increase in muscle strength. Stretching exercises can temporarily improve posture by elongating shortened muscles, but they were not more effective than cervical deep muscle flexion exercises because the proprioceptive motor control approach was not performed by strengthening the weakened muscles to maintain posture.

This study has several limitations. First, the study was conducted with 32 subjects to determine the effect of cervical deep neck flexion exercise on tension headaches and sleep disorders in patients with forward head posture. Thus, the results are difficult to generalize and interpret because of the small sample number, even though the sample size was statistically calculated. Second, posture change progresses slowly; therefore, long-term training intervention is required for continuous treatment. However, in this study, sufficient research was not conducted within the short period of 4 weeks, and follow-up was also not performed; therefore, the long-term effects are unknown. Additionally, as an appropriate comparison of the increase in muscle strength of the deep flexor muscles of the neck, it is suggested that an additional study of intervention through specific thoracic and lumbar motion methods other than through the cervical spine or the intervention for the increase in the strength of superficial muscles is necessary. Lastly, all patients had different severities of headache and sleep disorders, although the difference was not statistically significant. Further studies should divide these variables further and observe the differences.

## 5. Conclusions

In conclusion, on comparing both groups, it was found that the cervical deep muscle flexion exercise was helpful in relieving tension headache and improving sleep disorders in patients with forward head posture. Therefore, it is believed that the cervical deep muscle flexion exercise program will improve the quality of sleep and daily life by mitigating tension headaches and sleep disorders.

## Figures and Tables

**Table 1:** General characteristics of the participants (*n* = 32).

	EG (*n* = 16)	CG (*n* = 16)	* p *
Female sex, *n* (%) *	8 (50%)	5 (31.2%)	0.280
**Age (years)**	30.1 ± 2.7	31.0 ± 5.2	0.525
**Height (cm)**	164.4 ± 7.7	163.8 ± 8.2	0.843
**Weight (kg)**	61.8 ± 10.5	63.4 ± 9.9	0.656

Values are expressed as mean ± stand deviation except gender *. Abbreviations: EG, experimental group (cervical deep flexion exercise); CG, control group (stretching exercise).

**Table 2:** Variations in headache and sleep disorder (*n* = 32).

		EG (*n* = 16)	EG (*n* = 16)	*t*	*p*
**HDI** **(score)**	**Pre-test**	64.56 ± 13.38	57.88 ± 10.24	−2519	0.017
	**Post-test**	48.12 ± 10.78	57.38 ± 9.98		
	***t***	10.520	0.421		
	***p***	<0.001	0.679		
**PSQI-K** **(score)**	**Pre-test**	10.81 ± 1.97	10.50 ± 2.03	−3.316	0.002
	**Post-test**	7.56 ± 2.39	10.00 ± 2.25		
	***t***	10.070	1.936		
	***p***	<0.001	0.072		

Abbreviations: HDI, Headache Disability Inventory; PSQI, Pittsburgh Sleep Quality Index; EG, experimental group; CG, control group.

## Data Availability

The datasets generated during the current study are available from the corresponding author on reasonable request.

## References

[1] Hudson J.S., Ryan C.G. (2010). Multimodal group rehabilitation compared to usual care for patients with chronic neck pain: A pilot study. Man. Ther..

[2] Tepper M., Vollenbroek-Hutten M.M.R., Hermens H.J., Baten C. (2003). The effect of an ergonomic computer device on muscle activity of the upper trapezius muscle during typing. Appl. Ergon..

[3] Mekhora K., Liston C., Nanthavanij S., Cole J. (2000). The effect of ergonomic intervention on discomfort in computer users with tension neck syndrome. Int. J. Ind. Ergon..

[4] Nejati P., Lotfian S., Moezy A., Nejati M. (2015). The study of correlation between forward head posture and neck pain in Iranian office workers. Int. J. Occup. Med. Environ. Health.

[5] Petrofsky J.S., Laymon M., Alshammari F., Khowailed I.A., Lee H. (2017). Use of low level of continuous heat and Ibuprofen as an adjunct to physical therapy improves pain relief, range of motion and the compliance for home exercise in patients with nonspecific neck pain: A randomized controlled trial. J. Back Musculoskelet. Rehabil..

[6] Bryden L., Fitzgerald D. (2001). The influence of posture and alteration of function upon the craniocervical and craniofacial regions. Craniofacial Dysfunction and Pain: Manual Therapy, Assessment and Management.

[7] Szeto G.P., Straker L., Raine S. (2002). A field comparison of neck and shoulder postures in symptomatic and asymptomatic office workers. Appl. Ergon..

[8] Quek J., Pua Y.-H., Clark R.A., Bryant A.L. (2013). Effects of thoracic kyphosis and forward head posture on cervical range of motion in older adults. Man. Ther..

[9] Harrison D.E., Harrison D.D., Betz J.J., Janik T.J., Holland B., Colloca C.J., Haas J.W. (2003). Increasing the cervical lordosis with chiropractic biophysics seated combined extension-compression and transverse load cervical traction with cervical manipulation: Nonrandomized clinical control trial. J. Manip. Physiol. Ther..

[10] Fernández-De-Las-Peñas C., Pérez-de-Heredia M., Molero-Sánchez A., Miangolarra-Page J. (2007). Performance of the craniocervical flexion test, forward head posture, and headache clinical parameters in patients with chronic tension-type headache: A pilot study. J. Orthop. Sports Phys. Ther..

[11] Dodick D.W., Eross E.J., Parish J.M. (2003). Clinical, anatomical, and physiologic relationship between sleep and headache. J. Head Face Pain.

[12] Jull G.A. (2000). Deep cervical flexor muscle dysfunction in whiplash. J. Musculoskelet. Pain.

[13] Rubini E.C., Costa A.L., Gomes P.P.S.C. (2007). The Effects of Stretching on Strength Performance. Sports Med..

[14] Stovner L., Hagen K., Jensen R., Katsarava Z., Lipton R., Scher A., Steiner T., Zwart J.J.C. (2007). The global burden of headache: A documentation of headache prevalence and disability worldwide. Cephalalgia.

[15] Andersen L.L., Hansen K., Mortensen O.S., Zebis M.K. (2011). Prevalence and anatomical location of muscle tenderness in adults with nonspecific neck/shoulder pain. BMC Musculoskelet. Disord..

[16] Bronfort G., Assendelft W.J., Evans R., Haas M., Bouter L. (2001). Efficacy of spinal manipulation for chronic headache: A systematic review. J. Manip. Physiol. Ther..

[17] Lipchik G.L., Holroyd K.A., France C.R., Kvaal S.A., Segal D., Cordingley G.E., Rokicki L.A., McCool H.R. (1996). Central and peripheral mechanisms in chronic tension-type headache. Pain.

[18] Fernández-de-las-Peñas C., Alonso-Blanco C., Cuadrado M.L., Gerwin R.D., Pareja J.A. (2006). Trigger Points in the Suboccipital Muscles and Forward Head Posture in Tension-Type Headache. Headache J. Head Face Pain.

[19] Jung K.Y. (2006). Sleep and Headache. Korean J. Headache.

[20] Kelman L., Rains J.C. (2005). Headache and sleep: Examination of sleep patterns and complaints in a large clinical sample of migraineurs. Headache J. Head Face Pain.

[21] Lynch S.S., Thigpen C.A., Mihalik J.P., Prentice W.E., Padua D. (2010). The effects of an exercise intervention on forward head and rounded shoulder postures in elite swimmers. Br. J. Sports Med..

[22] Miller J., Gross A., D’Sylva J., Burnie S.J., Goldsmith C.H., Graham N., Haines T., Brønfort G., Hoving J.L. (2010). Manual therapy and exercise for neck pain: A systematic review. Man. Ther..

[23] Boyd-Clark L., Briggs C., Galea M.J.S. (2002). Muscle spindle distribution, morphology, and density in longus colli and multifidus muscles of the cervical spine. Spine.

[24] Cagnie B., Dirks R., Schouten M., Parlevliet T., Cambier D., Danneels L. (2011). Functional reorganization of cervical flexor activity because of induced muscle pain evaluated by muscle functional magnetic resonance imaging. Man. Ther..

[25] Falla D.L., Jull G.A., Hodges P.W. (2004). Patients With Neck Pain Demonstrate Reduced Electromyographic Activity of the Deep Cervical Flexor Muscles During Performance of the Craniocervical Flexion Test. Spine.

[26] Randløv A., Østergaard M., Manniche C., Kryger P., Jordan A., Heegaardand S., Holm B. (1998). Intensive dynamic training for females with chronic neck/shoulder pain. A randomized controlled trial. Clin. Rehabil..

[27] Nemmers T.M., Miller J.W., Hartman M.D. (2009). Variability of the Forward Head Posture in Healthy Community-dwelling Older Women. J. Geriatr. Phys. Ther..

[28] Comerford M., Mottram S. (2012). Kinetic Control-e-Book: The Management of Uncontrolled Movement.

[29] Comerford M., Mottram S.L. (2011). Diagnosis of Uncontrolled Movement, Subgroup Classification and Motor Control Retraining of the Neck.

[30] Jesus F.M., Ferreira P.H., Ferreira M.L. (2008). Ultrasonographic measurement of neck muscle recruitment: A preliminary investigation. J. Man. Manip. Ther..

[31] Evjenth O. (2001). Autostretching: The Complete Manual of Specific Stretching.

[32] Jacobson G.P., Ramadan N.M., Aggarwal S.K., Newman C.W. (1994). The Henry Ford Hospital Headache Disability Inventory (HDI). Neurology.

[33] Sohn S.I., Kim D.H., Lee M.Y., Cho Y.W. (2012). The reliability and validity of the Korean version of the Pittsburgh Sleep Quality Index. Sleep Breath.

[34] Buysse D.J., Reynolds C.F., Monk T.H., Berman S.R., Kupfer D.J. (1989). The Pittsburgh Sleep Quality Index: A new instrument for psychiatric practice and research. Psychiatry Res..

[35] Lee G.-H. (2007). Postural Control in the Patients with Chronic Tension-Type Headache. J. Korean Neurol. Assoc..

[36] Chae Y.-W., Lee H.-M. (2009). The effect of craniocervical exercise on tension-type headache. J. Korean Phys. Therapy.

[37] Martin-Herrero C., Rodrigues de Souza D.P., Alburquerque-Sendin F., Ortega-Santiago R., Fernández-de-Las-Peñas C. (2012). Myofascial trigger points, pain, disability and quality of sleep in patients with chronic tension-type headache: A pilot study. Rev. Neurol..

[38] Peck D., Buxton D., Nitz A. (1984). A comparison of spindle concentrations in large and small muscles acting in parallel combinations. J. Morphol..

[39] Dugailly P.-M., Sobczak S., Moiseev F., Sholukha V., Salvia P., Feipel V., Rooze M., Jan S.V. (2011). Musculoskeletal modeling of the suboccipital spine: Kinematics analysis, muscle lengths, and muscle moment arms during axial rotation and flexion extension. Spine.

[40] Canseco J.A., Schroeder G.D., Patel P.D., Grasso G., Chang M., Kandziora F., Vialle E.N., Oner F.C., Schnake K.J. (2021). Regional and experiential differences in surgeon preference for the treatment of cervical facet injuries: A case study survey with the AO Spine Cervical Classification Validation Group. Eur. Spine J..

[41] Bogduk N. (1992). The anatomical basis for cervicogenic headache. J. Manip. Physiol. Ther..

[42] Bendtsen L. (2000). Central sensitization in tension-type headache—Possible pathophysiological mechanisms. Cephalalgia.

[43] Harris K.D., Heer D.M., Roy T.C., Santos D.M., Whitman J.M., Wainner R.S. (2005). Reliability of a Measurement of Neck Flexor Muscle Endurance. Phys. Ther..

[44] Provini F., Vetrugno R., Lugaresi E., Montagna P. (2006). Sleep-related breathing disorders and headache. Neurol. Sci..

[45] Rains J.C., Poceta J.S. (2006). Headache and Sleep Disorders: Review and Clinical Implications for Headache Management. Headache J. Head Face Pain.

[46] Kogler A., Lindfors J., Ödkvist L., Ledin T. (2000). Postural stability using different neck positions in normal subjects and patients with neck trauma. Acta Oto-Laryngol..

